# A Method of Assessment of Human Natural Killer Cell Phenotype and Function in Whole Blood

**DOI:** 10.3389/fimmu.2020.00963

**Published:** 2020-05-20

**Authors:** Marisa Market, Gayashan Tennakoon, Juliana Ng, Marlena Scaffidi, Christiano Tanese de Souza, Michael A. Kennedy, Rebecca C. Auer

**Affiliations:** ^1^University of Ottawa, Ottawa, ON, Canada; ^2^Cancer Therapeutics Program, Ottawa Hospital Research Institute, Ottawa, ON, Canada; ^3^Department of Surgery, University of Ottawa, The Ottawa Hospital, Ottawa, ON, Canada

**Keywords:** natural killer cells, whole blood, flow cytometry, cytokine stimulation, interferon gamma, NKG2D

## Abstract

The majority of data on human Natural Killer (NK) cell phenotype and function has been generated using cryopreserved peripheral blood mononuclear cells (PBMCs). However, cryopreservation can have adverse effects on PBMCs. In contrast, investigating immune cells in whole blood can reduce the time, volume of blood required, and potential artefacts associated with manipulation of the cells. Whole blood collected from healthy donors and cancer patients was processed by three separate protocols that can be used independently or in parallel to assess extracellular receptors, intracellular signaling protein phosphorylation, and intracellular and extracellular cytokine production in human NK cells. To assess extracellular receptor expression, 200 μL of whole blood was incubated with an extracellular staining (ECS) mix and cells were subsequently fixed and RBCs lysed prior to analysis. The phosphorylation status of signaling proteins was assessed in 500 μL of whole blood following co-incubation with interleukin (IL)-2/12 and an ECS mix for 20 min prior to cell fixation, RBC lysis, and subsequent permeabilization for staining with an intracellular staining (ICS) mix. Cytokine production (IFNγ) was similarly assessed by incubating 1 mL of whole blood with PMA-ionomycin or IL-2/12 prior to incubation with ECS and subsequent ICS antibodies. In addition, plasma was collected from stimulated samples prior to ECS for quantification of secreted IFNγ by ELISA. Results were consistent, despite inherent inter-patient variability. Although we did not investigate an exhaustive list of targets, this approach enabled quantification of representative ECS surface markers including activating (NKG2D and DNAM-1) and inhibitory (NKG2A, PD-1, TIGIT, and TIM-3) receptors, cytokine receptors (CD25, CD122, CD132, and CD212) and ICS markers associated with NK cell activation following stimulation, including signaling protein phosphorylation (p-STAT4, p-STAT5, p-p38 MAPK, p-S6) and IFNγ in both healthy donors and cancer patients. In addition, we compared extracellular receptor expression using whole blood vs. cryopreserved PBMCs and observed a significant difference in the expression of almost all receptors. The methods presented permit a relatively rapid parallel assessment of immune cell receptor expression, signaling protein activity, and cytokine production in a minimal volume of whole blood from both healthy donors and cancer patients.

## Introduction

Natural Killer (NK) cells, first identified by Kiessling et al. in 1975, are cytotoxic lymphocytes that play a critical role in the innate immune response through the destruction of stressed, infected, or cancerous cells (1). Defective NK cell function has been linked to autoimmune and infectious diseases as well as cancer (2–6). Our investigations focus on understanding the suppression of NK cells following surgery in cancer patients and the impact of immunosuppression on metastasis. Specifically, our lab and others have shown that postoperative defects in NK cell cytotoxicity and IFNγ production contribute to increased metastasis in models of surgical stress (7–9). Our initial observations of this suppressed phenotype were in cryopreserved peripheral blood mononuclear cells (PBMCs); however, we have also observed this phenomenon in whole blood. We then developed protocols that can be used in parallel to assess the phenotype, intracellular signaling following cytokine stimulation, and cytokine production of immune cells, and as an example, in this paper we highlight its implementation for our ongoing research investigating NK cells in cancer patients.

For practical reasons, the majority of the data on human NK cells has been generated using PBMCs. For instance, cryopreservation allows for running batched samples simultaneously as well as logistical flexibility for the storage and shipment of samples between research facilities (10). Using this approach, the study of cryopreserved PBMCs through functional and phenotypic assays has yielded a great deal of understanding about the role of NK cell function in disease. However, the use of cryopreserved PBMCs in immunologic studies is associated with adverse effects on cell populations/certain cell markers and altered gene expression (11–13). As a result, our understanding of NK cells may benefit in certain circumstances from investigations of non-cryopreserved cells.

In trying to assess the mechanism of NK cell dysfunction in cancer patients in the context of surgery, we sought to assess key markers and intracellular pathways associated with this dysfunctional NK cell phenotype. We investigated upstream receptor expression and subsequent signaling protein phosphorylation in order to elucidate the mechanism of NK cell suppression. NK cells do not undergo clonal selection, they instead express a limited number of germline-encoded receptors (14). NK cell activating receptors recognize pathogen-derived antigens as well as stress-induced ligands in what is termed the “induced-self recognition model” (15–17). These activating signals are antagonized by inhibitory receptors that recognize constitutively expressed self-molecules or inhibitory checkpoint proteins (15, 16). We sought to assess the expression levels of the activating receptors NKG2D and DNAM-1 and the inhibitory receptors NKG2A, PD-1, TIGIT, and TIM-3. In addition to these receptors, NK cells also express a plethora of cytokine receptors, including interleukin (IL)-2R and IL-12R (18). NK cell activity is thus regulated by the integration of activating and inhibitory ligands through these many receptors, which results in phosphorylation and signal transduction through signaling proteins such as STAT4, STAT5, p38 MAPK, and S6 (9, 19–23). This culminates in the regulation of transcription factor activity that controls the transcription of cytokines such as IFNγ and cytotoxic proteins, including granzymes and perforin (24, 25). In characterizing the perioperative NK cell phenotype, we found it challenging to assess phosphorylation status in cryopreserved PBMCs. As a solution, we considered the use of whole blood, which proved to be far superior. In the troubleshooting process we also discovered a discrepancy between the phenotypes observed in cryopreserved PBMCs vs. whole blood staining. The successes we experienced by using whole blood samples, compared to cryopreserved PBMCs, prompted us to continue using whole blood samples for assessment of NK cell activity and develop a series of easily implemented, standardized protocols that enable a comprehensive investigation of NK phenotype and function.

There is a paucity of studies investigating immune cell function from whole blood (26). We posit that such studies would avoid the adverse effects of cryopreservation and provide more biologically relevant results in some circumstances. For example, investigating protein phosphorylation states by flow cytometry is difficult in cryopreserved samples due to the poor signal to noise ratio of the target protein compared to investigations in whole blood samples (27). Many of these limitations can be overcome by staining directly in whole blood, which also allows for simpler and faster protocols that require minimal manipulation of the cells of interest and therefore support the biological relevance of the results. A limitation of whole blood assays includes having to process patient samples immediately and therefore they cannot be tested simultaneously, which could lead to greater inter-assay variability. However, technical expertise, appropriate controls, and validated standard operating procedures can be implemented to help mitigate this limitation.

Comparisons of immunologic assays using cryopreserved PBMCs and whole blood samples have previously been reported and is not the focus of our report (24, 25, 28). Here we sought to highlight the feasibility and advantages of using whole blood samples as a strategy for phenotypic and functional assessments in NK cells. As a proof of concept, we show the utilization of these protocols in our ongoing research. We explored the differential expression of phenotypic receptors necessary for NK activity and phosphorylation of downstream signaling molecules in healthy donors and cancer patients using whole blood. Finally, NK cell function was investigated by quantifying intracellular and extracellular IFNγ by flow cytometry and ELISA following stimulation with PMA-ionomycin or IL-2/IL-12. We show that assaying cryopreserved cells results in altered NK cell phenotype in human patients as compared to whole blood analysis. In addition, we outline in detail novel whole blood protocols that can be used in parallel to assess immune cell receptor expression, signaling protein phosphorylation, and cytokine production. Although developed to assess NK cell activity in the perioperative period, these protocols could be used to assess other immune cell phenotypes in other pathological conditions.

## Materials and Equipment

Equipment required includes a 37°C incubator, a 37°C water bath, a centrifuge, an ELISA Microplate Reader, and a flow cytometer (LSR Fortessa). Sodium-heparin tubes (BD Vacutainer® Cat #367878/367874) were used to collect healthy donor and patient blood samples. Stimulation reagents included PMA (phorbol 12-myristate 13-acetate; Sigma Aldrich Cat #P8139), Ionomycin (Sigma-Aldrich Cat #I9657-1MG), recombinant human IL-2 (Tecin Teceleukin) and recombinant human IL-12 (R&D System Cat #219-IL005). Reagents used in staining protocols include: Phosphate Buffered Saline (PBS), BD Golgiplug (Brefaldin A) (Cat #51-2301K2), BD FACS Lyse/ Fix Buffer (Cat #558049), deionized/ distilled H_2_O, Flow Buffer (PBS + 2.5g BSA + 0.5M EDTA), BD Perm III Buffer (Cat #558050), and 1% Paraformaldehyde. Extracellular IFNγ was quantified using the R&D Quantikine Human IFNγ ELISA (Cat #DIF50). Antibodies used for FACS staining are listed in Table 1.

**Table 1:** Antibodies used in whole blood panels.

**Antibody**	**Vendor**	**Cat #**	**Clone**
CD3 FITC (mouse)	Invitrogen	11-0039-41	HIT3a
CD56 BV421 (mouse)	BD biosciences	562751	NCAM16.2
CD16 BV650 (mouse)	BD biosciences	563692	3G8
CD14 APC-Cy7 (mouse)	BD biosciences	557831	MϕP9
CD45 AF700 (mouse)	BD biosciences	560566	HI30
Fixable viability dye BV510	BD biosciences	564406	–
IFNγ APC (mouse)	Invitrogen	17-7319-82	4S.B3
CD25 PE-Cy7 (mouse)	BD biosciences	557741	M-A251
CD122 PE (mouse)	BD biosciences	554522	Mik-β2
CD132 APC (rat)	Biolegend	338607	TUGh4
p-STAT5 PE-Cy7 (pY694) (mouse)	BD biosciences	560117	47/Stat5
CD212 BV786 (mouse)	BD biosciences	744207	2.4E6
Mouse BV786 IgG1	BD biosciences	563330	X40
p-STAT4 PE (pY693) (mouse)	BD biosciences	558249	38/p-Stat4
NKG2D BV650 (mouse)	BD biosciences	563408	1D11
NKG2A PE (mouse)	R&D Systems	FAB1059P-025	131411
BV786 TIM-3 (mouse)	BD biosciences	742857	7D3
PE-Cy7 DNAM-1 (mouse)	BioLegend	338315	11A8
APC TIGIT (mouse)	BioLegend	372705	A15153G
PD-1 PerCP-Cy5.5 (mouse)	BioLegend	329913	EH12.2H7
S6 PE (pS235/236) (mouse)	BD biosciences	560433	NF-548
p38 MAPK APC (pThr180, Tyr 182) (mouse)	Invitrogen	17-9078-42	4NIT4KK
Mouse PE IgG2a	BioLegend	400214	MOPC-173
Mouse APC IgG2A	BioLegend	400219	MOPC-173
Mouse PerCP-Cy5.5 IgG1	BioLegend	400149	MOPC-21
Mouse APC IgG1	Biolegend	400119	MOPC-21
Mouse PE-Cy7 IgG1	BD biosciences	557872	MOPC-21
Mouse PE IgG2b	Invitrogen	12-4732-41	eBMG2b
Mouse BV650 IgG1	BD biosciences	563231	X40
Rat APC IgG2b	Biolegend	400611	RTK4530

## Methods

This protocol was approved by the Ottawa Health Science Research Ethics Board. All subjects gave written informed consent in accordance with the Declaration of Helsinki. Eligible patients were >18 years of age and had a planned surgical resection of the primary or metastatic tumor (cancer patients) or healthy donors who volunteered to participate. Exclusion criteria included a history of active viral or bacterial infection or known HIV or Hepatitis B or C, autoimmune diseases, or use of immunosuppressive medications.

### Protocol 1—Extracellular Receptor Staining (Supplementary Material; Figure 1A)

Objective: Assess cell surface receptor expression in Natural Killer cells from whole blood.

**Figure 1:**
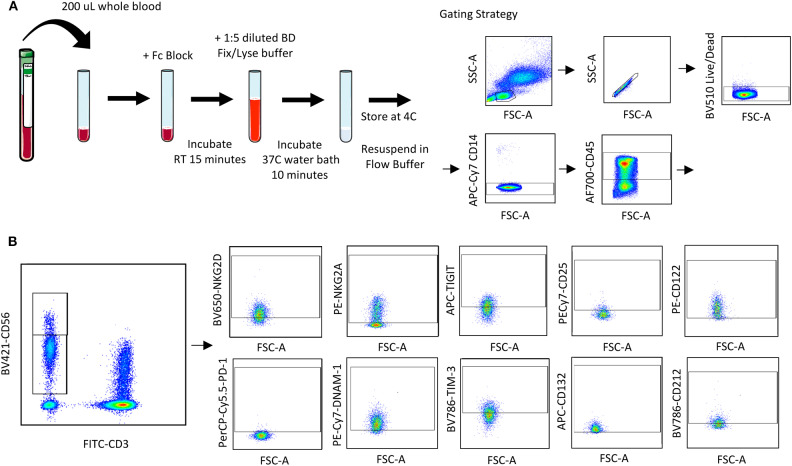
Whole blood receptor methodology and gating strategy. Whole blood was collected from patients and 200 μL was aliquoted per receptor panel. After a 15-min incubation with the receptor panel, lyse/fix buffer was added and incubated for 10 min before blood was spun down, cells were washed and resuspended to be assessed by flow cytometry. The lymphocyte population was gated on before excluding doublets and dead cells **(A)**. CD45^+^ CD14^−^ CD56^Bright/Dim^ CD3^−^ cells were gated on to assess activating/inhibitory receptor and cytokine receptor expression based on isotype staining **(B)**.

Blood was collected from healthy donors and cancer surgery patients at baseline and on POD1. Two hundred microliters of whole blood was aliquoted per flow cytometry panel into a 15 mL conical tube. Forty microliters of extracellular staining (ECS) mix was added and mixed by pipetting. Blood was incubated for 15 min at room temperature (RT) prior to adding 4 mL of BD FACS lyse/fix buffer (BD Cat #558049; 1:5 dilution with diH_2_O). Tube were shaken vigorously to ensure red blood cell (RBC) lysis. Tubes were incubated for 10 min in a 37°C water bath and centrifuged at 500 g for 8 min. Supernatant was carefully aspirated, and the cell pellet was resuspended in 1 mL flow buffer (FB; 500 mL PBS, 2.5 g Bovine serum albumin, 1 mL 0.5 M EDTA). Cells were centrifuged at 500 g for 5 min and supernatant aspirated prior to resuspending in 200 μL 1% paraformaldehyde (PFA). Samples were stored at 4°C for up to 72 h prior to acquisition and analysis by flow cytometry. At least 2,500 events were collected, gating on CD56^+^CD3^−^ NK cells.

### Protocol 2—Intracellular Signaling Protein Phosphorylation Staining (Supplementary Material; **Figure 4A**)

Objective: Assess signaling protein/ transcription factor phosphorylation in response to stimuli in Natural Killer cells from whole blood.

Blood was collected from healthy donors and cancer surgery patients at baseline and on POD1. Five hundred microliters of whole blood was aliquoted per flow cytometry panel into a new sodium-heparin tube. Control (PBS) or IL-2/12 stimulation (400 U/20 ng/mL) and 40 μL of ECS mix was added and mixed by pipetting. Blood was incubated for 20 min in a 37°C water bath and then transferred to a 15 mL conical tube prior to adding 10 mL of BD FACS lyse/fix buffer (1:5 dilution with diH_2_O). Tubes were shaken vigorously to ensure RBC lysis. Tubes were incubated for 10 min in a 37°C water bath and centrifuged at 500 g for 8 min. Supernatant was carefully aspirated, and the cell pellet was resuspended in 1 mL FB. Cells were centrifuged at 500 g for 5 min and supernatant was aspirated prior to resuspending in 500 μL chilled BD Perm III buffer. Cells were incubated on ice in the dark for 30 min and centrifuged at 300 g for 10 min. Supernatant was aspirated and the pellet was resuspended in 400 μL FB; 200 μL per well was then transferred into a 96 well v-bottom plate and centrifuged at 500 g for 5 min. The plate was decanted, and cells were resuspended in 200 μL of appropriate intracellular staining (ICS) mix and incubated at RT in the dark for 1 h. The plate was spun at 500 g for 5 min and cells were resuspended in 1% PFA. Samples were stored at 4°C for up to 72 h prior to acquisition and analysis by flow cytometry. At least 2, 500 events were collected, gating on CD56^+^CD3^−^ NK cells.

### Protocol 3—Intracellular IFNγ Staining (Supplementary Material; **Figure 5A**)

Objective: Quantify intracellular IFNγ production as a measure of activity in Natural Killer cells from whole blood.

Blood was collected from healthy donors and cancer surgery patients at baseline and on POD1. One milliliter of whole blood was aliquoted per flow cytometry panel into a new sodium-heparin tube. Whole blood was incubated with PBS (control) or PMA-ionomycin (50 ng/750 ng/mL) for 5 h or IL-2/12 (400 U/20 ng/mL) for 24 h at 37°C. 10 μg/mL Golgiplug (Brefaldin A) per tube was added, tubes were inverted 10 times to mix, and incubated at 37°C for the remaining 2 h of each incubation. Six hundred microliter of whole blood was then collected in an Eppendorf tube, centrifuged at 500 g 5 min, and plasma was collected and stored at −80°C for an IFNγ ELISA. The remaining 400 μL of whole blood was transferred to a new 15 mL conical tube and incubated with Fc block (Human Trustain; Biolegend Cat #422302) for 5 min at RT. The ECS mix was then added and mixed by pipetting. Blood was incubated for 15 min at RT prior to adding 20 mL of BD FACS lyse/fix buffer (1:5 dilution with diH_2_O). Tube were shaken vigorously to ensure RBC lysis. Tubes were incubated for 10 min in a 37°C water bath and centrifuged at 500 g for 8 min. Supernatant was carefully aspirated, and the cell pellet was resuspended in 1 mL FB. Cells were centrifuged at 500 g for 5 min and supernatant was aspirated prior to resuspending in 500 μL chilled BD Perm III buffer (BD Cat #558050). Cells were incubated on ice in the dark for 30 min and centrifuged at 300 g for 10 min. Supernatant was aspirated and the pellet was resuspended in 400 μL FB; 200 μL per well was then transferred into a 96-well v-bottom plate and centrifuged at 500 g for 5 min. The plate was decanted, and cells were resuspended in 200 μL of appropriate ICS mix and incubated at 4°C for 30 min in the dark for 1 h. The plate was spun at 500 g for 5 min and cells were resuspended in 1% PFA. Samples were stored at 4°C for up to 72 h prior to acquisition and analysis by flow cytometry. At least 2, 500 events were collected, gating on CD56^+^CD3^−^ NK cells.

### Extracellular IFNγ Quantification (Supplementary Material; **Figure 6A**)

Objective: Quantify extracellular IFNγ production as a measure of activity in plasma from whole blood.

The R&D Quantikine® ELISA Human IFNγ Immunoassay (Cat #DIF50) was used to quantify extracellular IFNγ from cell culture supernatant and patient plasma. Samples were thawed at RT and either run undiluted or at a dilution of 5x or 10x with appropriate buffer. This assay was run following the R&D Quantikine® ELISA protocol. The minimum detectable dose (MDD) for the assay is <8.0 pg/mL.

## Data Analysis

Descriptive statistics were used to summarize data collected on extracellular receptors, phospho-signaling proteins, and IFNγ production [median with interquartile range (IQR)]. Wilcoxon matched-pairs signed rank test was used to determine if there were significant changes in receptor expression (percentage and MFI) between cryopreserved and whole blood samples. The level for statistical significance was set a priori at ≤ 0.05 (^*^p ≤ 0.05, ^**^p ≤ 0.005, ^***^p ≤ 0.0005, ^****^p ≤ 0.00005). All statistical analyses were performed using Prism 8.

## Results

### Protocol 1—Extracellular Receptor Staining

#### Quantifying NK Cell Surface Receptors in Whole Blood

**Figure 2:**
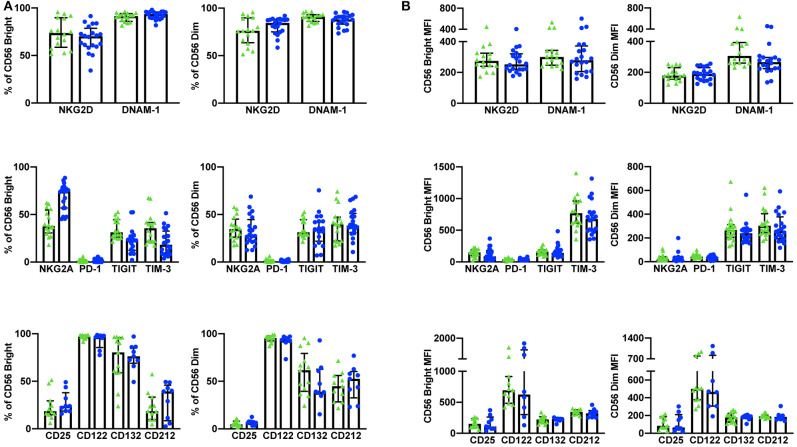
Whole blood extracellular surface receptor expression. The percentage of CD56^Bright/Dim^ CD3^−^ cells expressing activating receptors (NKG2D and DNAM-1), inhibitory receptors (NKG2A, PD-1, TIGIT, and TIM-3), and cytokine receptor subunits (CD25, CD122, CD132, and CD212) was assessed in healthy donors (*n* = 29) and cancer patients (*n* = 31) **(A)**. The relative level of expression (MFI) of the same activating/inhibitory/cytokine receptors was also assessed in CD56^Bright/Dim^ CD3^−^ cells **(B)**. Shown are the median values ± IQR.

Using a 10 color flow cytometry panel, we assessed the surface expression of six NK cell receptors, which are known to activate (NKG2D and DNAM-1) or inhibit (NKG2A, PD-1, TIGIT, and TIM-3) NK cell effector functions in a cohort of 16 healthy donors and 20 cancer patients (Table 2). Using a nine color flow cytometry panel, we similarly assessed whether the expression of IL-2/12 receptor subunits [CD25 (α), CD122 (β), CD132 (γ), and CD212 (β1)] could be detected in 13 healthy donors and 11 cancer patients (Table 2). We assessed the percentage of positive cells as well as the relative expression level (median fluorescence intensity/MFI) of both activating/inhibitory and cytokine receptors in CD56^Bright^CD3^−^ and CD56^Dim^CD3^−^ NK cells using the indicated gating strategy (Figure 1). Gates were set based on matched isotype controls (Supplementary Figure 1). We were able to assess both activating/inhibitory and cytokine receptor expression using this whole blood protocol (Figure 2 and Supplementary Table 1).

**Table 2:** Whole blood patient demographics.

**Category**	**Subcategory**	**Healthy donors**	**Patients**
Total (*n*)		40	39
Sex	Male	16	21
	Female	24	18
Patient age	<60 years	24	11
	60–69 years	9	19
	>70 years	7	9
Cancer type	Prostate	–	11
	Lung	–	7
	Colorectal	–	6
	Renal	–	3
	Ovarian	–	3
	Sarcoma	–	3
	Pancreatic	–	1
	Parathyroid	–	1
	Neuroendocrine	–	1
	Endometrial	–	1
	Duodenal	–	1
	Uterine	–	1
Staging	I	–	14
	II	–	7
	III	–	15
	IV	–	3

*Summary of patient information including sex, age, cancer type, and cancer staging for patient samples used to quantify receptor expression, phosphorylation status, and IFNγ production using whole blood*.

#### Discrepancies Between Whole Blood and Cryopreserved NK Cell Surface Receptors

**Figure 3:**
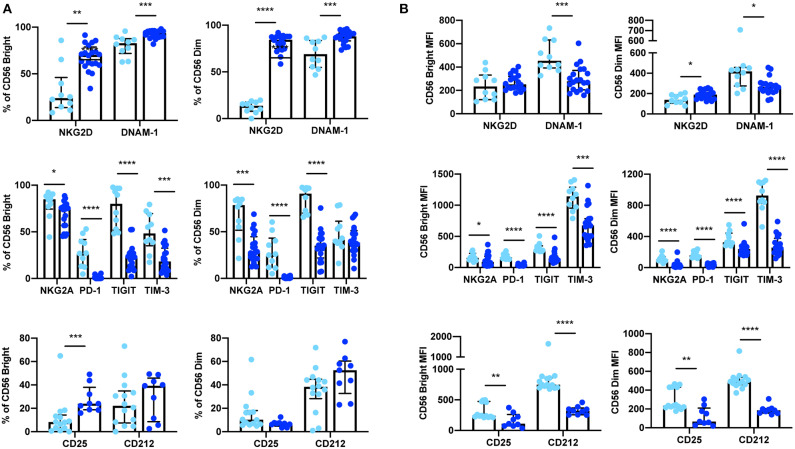
Expression of extracellular surface receptors differs between cryopreserved PBMCs and whole blood samples. A significant difference was observed between the percentage of CD56^Bright/Dim^ CD3^−^ cells expressing all activating and inhibitory receptors (cryopreserved *n* = 10, whole blood *n* =20), as well as the cytokine receptor subunits CD25 and CD212 (cryopreserved *n* = 14, whole blood *n* = 11) **(A)**. In addition, a significant difference was also observed in the relative expression levels (MFI) of these receptors in both CD56^Bright^ and CD56^Dim^ populations **(B)**. Shown are the median values ± IQR. The Mann-Whitney test was used to assess statistical significance. *p* ≤ 0.05 (*p ≤ 0.05, **p ≤ 0.005, ***p ≤ 0.0005, ****p ≤ 0.00005).

In addition to whole blood we also assessed the expression of NKG2D, DNAM-1, PD-1, TIGIT, and TIM-3 (*n* = 10) and CD25 and CD212 (*n* = 14) in NK cells from cryopreserved PBMCs (Table 3). After Ficoll density centrifugation, PBMCs were isolated, washed, and stored in liquid nitrogen in 90% FBS 10% DMSO. We followed a standard protocol whereby PBMCs were thawed, rested overnight, and stained using a 10 color (activating/inhibitory receptors) or a nine color (cytokine receptors) flow cytometry panel (29–32) PBMC viability and yield after thawing were ≥73 and ≥50%, respectively. At least 2,500 events were collected, gating on CD56^+^CD3^−^ NK cells (gating strategy shown in Supplementary Figure 2). Consistent with previous publications, we found significant differences between the percentage of positive cells and receptor MFI in cryopreserved vs. whole blood NK cells (33, 34) (Figure 3, Supplementary Table 2).

**Table 3:** Cryopreserved PBMC patient demographics.

**Category**	**Subcategory**	**Patients**
Total (n)		19
Gender	Male	13
	Female	6
Patient Age	<60 years	5
	60-69 years	9
	>70 years	5
Cancer Type	Prostate	8
	Lung	8
	Colorectal	2
	Esophageal	1
Staging	I	4
	II	5
	III	7
	IV	2
	Unknown	1

*Summary of patient information including sex, age, cancer type, and cancer staging for patient samples used to quantify activating/inhibitory/cytokine receptors using cryopreserved PBMCs*.

### Protocol 2—Intracellular Signaling Protein Phosphorylation Staining

#### Detecting NK Cell Cytokine Signaling in Whole Blood

**Figure 4:**
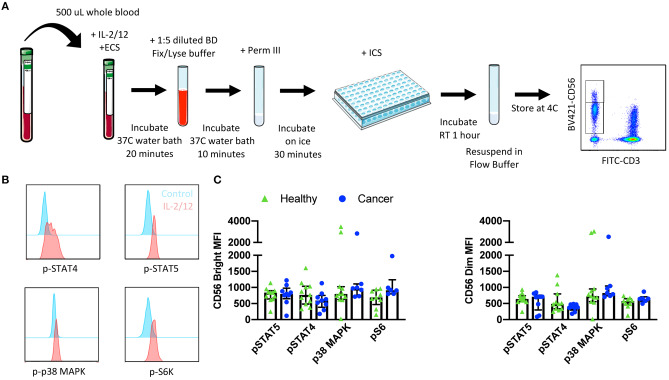
Whole blood signaling protein methodology, gating strategy, and quantification. Whole blood was collected from patients and 500 μL was aliquoted per receptor panel. After a 15-min incubation with IL-2/12 stimulation and the desired receptor panel, lyse/fix buffer was added and incubated for 10 min before blood was spun down. Cells were washed, resuspended in Perm III Buffer, and incubated on ice for 30 min before being spun down and resuspended in an intracellular staining mix. Cells were then incubated for an additional hour at room temperature prior to being resuspended and assessed by flow cytometry **(A)**. The lymphocyte population was gated on before excluding doublets and dead cells. CD45^+^ CD14^−^ CD56^Bright/Dim^ CD3^−^ cells were gated on to assess signaling protein phosphorylation **(B)**. The relative level of expression (MFI) of phospho-proteins STAT5, STAT4, p38 MAPK, and S6 was assessed in healthy donor (*n* = 13) and cancer patient (*n* = 9) samples **(C)**. Shown are the median values ± IQR.

The phosphorylation of signaling proteins downstream of IL-2/12 receptors was assessed using two 7 color flow cytometry panels in 13 healthy donors and nine cancer patients. Phospho-specific antibodies against STAT5, STAT4, p38 MAPK, and S6 were used to determine the relative phosphorylation of (MFI) the relative phosphorylation (MFI) in CD56^Bright^CD3^−^ and CD56^Dim^CD3^−^ NK cells (Figure 4B). We were able to quantify the phosphorylation of these signaling molecules in response to IL-2/12 stimulation in both healthy donors and cancer patients (Figure 4C, Supplementary Table 3).

### Protocol 3—Intracellular IFNγ Staining

#### Quantifying NK Cell Responsiveness to Cytokine Stimulation

**Figure 5:**
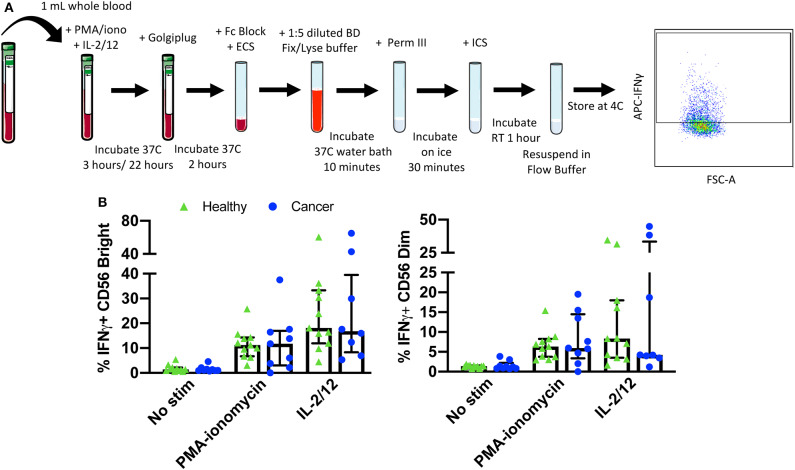
Whole blood secretory cytokine methodology, gating strategy, and quantification. Whole blood was collected from patients and 1 mL was aliquoted per stimulatory condition. Whole blood was stimulated for 24 h in the presence of PMA-ionomycin or IL-2/12. At 22 h post-stimulation, Golgiplug was added to whole blood and incubated for an additional 2 h. At 24 h post-stimulation, whole blood was incubated for 15 min with an extracellular staining mix. Lyse/fix buffer was then added and incubated for 10 min before blood was spun down. Cells were washed, resuspended in Perm III Buffer, and incubated on ice for 30 min before being spun down and resuspended in an intracellular staining mix. Cells were then incubated for an additional 30 min at 4°C prior to being resuspended and assessed by flow cytometry **(A)**. The lymphocyte population was gated on before excluding doublets and dead cells. CD45^+^ CD14^−^ CD56^Bright/Dim^ CD3^−^ cells were gated on to assess intracellular IFNγ based on unstimulated controls. The percentage of CD56^Bright/Dim^ CD3^−^ cells expressing INFγ after stimulation was assessed in healthy donors (*n* = 11) and cancer patients (*n* = 9) **(B)**. Shown are the median values ± IQR.

**Figure 6:**
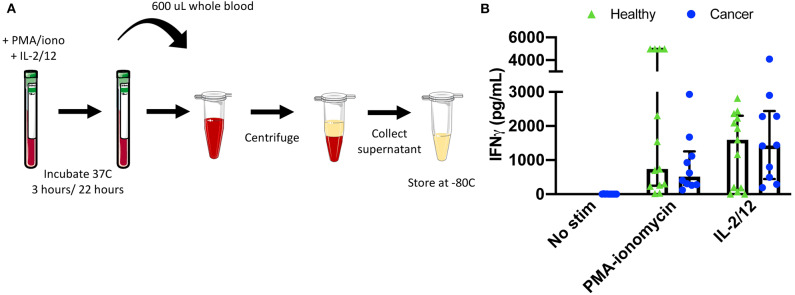
Plasma collection methodology for secretory cytokine quantification by ELISA. After incubation in the absence of stimulation or with either PMA-ionomycin or IL-2/12 stimulation for 24 h, 600 μL of whole blood was collected and spun down prior to being collected and stored at −80°C for subsequent use in a human IFNγ ELISA **(A)**. Extracellular IFNγ was quantified in response to stimuli in healthy donors (*n* = 13) and cancer patients (*n* = 10) **(B)**. Shown are the median values ± IQR.

Finally, NK cell activity was quantified by measuring both intracellular and extracellular IFNγ production in response to stimulation with either PMA-ionomycin (a receptor-independent stimulator of cytokine production) or IL-2/12. A six color flow cytometry panel and the indicated gating strategy was used to assess the percentage of CD56^Bright^CD3^−^ and CD56^Dim^CD3^−^ NK cells producing IFNγ in 11 healthy donors and nine cancer patients (Figure 5A). Gates were set based on matched unstimulated controls (Supplementary Figure 1). The healthy donor and cancer patient populations produced intracellular IFNγ in response to both stimuli (Figure 5B, Supplementary Table 4). In addition, extracellular IFNγ was quantified in 13 healthy donors and 10 cancer patients from plasma collected prior to intracellular staining (Figure 6B, Supplementary Table 4).

Next, we investigated whether increased IFNγ production could be correlated with phosphorylation of upstream signaling proteins. Since STAT4 is phosphorylated in response to IL-12 receptor binding and contributes to IFNγ production we investigated the correlation of pSTAT4 MFI and IFNγ MFI in CD56^Bright^ and CD56^Dim^ NK cells. Notably, we observed a moderate correlation between pSTAT4 and IFNγ MFI in both CD56^Bright^ (*R*^2^ = 0.2819, *p* < 0.05) and CD56^Dim^ (*R*^2^ = 0.2148, *p* < 0.05) populations, suggesting that upstream phosphorylation events are correlated with cytokine production despite differences in sample preparation (Supplementary Figure 3). The strength of this relationship may be impacted by the heterogeneity of patient samples as well as the small patient population being assessed. However, similar relationships have been reported in the literature to support causal relationships in assessing lymphocyte dysfunction (35).

Granulocytes are large lymphocytes that contain cytoplasmic granules and include neutrophils, basophils, and eosinophils, with neutrophils being the most abundant leukocyte in human blood (36). Neutrophils have been shown to have pleotropic effects on NK cells ranging from inducing NK cell licensing to inhibiting proliferation and IFNγ production, to enhancing cytotoxic activity (37, 38). The Ficoll separation of PBMCs allows for the removal of higher density neutrophils. However, in using whole blood for phenotypic and functional assays granulocytes are present at biologically relevant concentrations. In order to discern whether these cells were having an impact on NK cell cytokine production, we quantified the percentage of CD14^−^FSC-A^hi^SSC-A^hi^ cells and plotted this against intracellular IFNγ in the same 11 healthy donors and nine cancer patients. We did not find a correlation between the two, suggesting that in these whole blood protocols, IFNγ production is not influenced by the presence of granulocytes (Supplementary Figure 4).

## Advantages

These assays require minimal volumes of blood (200 μL-1 mL per sample) as compared to methods employing cryopreserved PBMCs (~30–40 mL). This allows for the simultaneous assessment of many targets and the use of whole blood from one patient for multiple assays. In addition, patients may be more likely to consent to blood draws for research that requires minimal blood volumes. These assays can be used to effectively measure extracellular and intracellular targets in healthy as well as disease states (cancer). Here we have assessed CD56^Bright^CD3^−^ and CD56^Dim^CD3^−^ NK cell phenotype and function; however, the use of whole blood allows for the assessment of any immune cell, including neutrophils which would otherwise the excluded in PBMC isolation protocols. Finally, a discrepancy can be seen between extracellular targets measured on NK cells using whole blood vs. cryopreserved PBMCs. We believe that assessing immune cell phenotypes using whole blood may be more biologically relevant as these protocols minimize the time between blood draw and cell staining and reduce the manipulation of cells that may otherwise impact target expression.

## Limitations

Although the protocol described in this paper features several technical and scientific advantages, it is important to note the potential limitations. However, through the use of appropriate controls (unstained, isotype, fluorescence minus one, “healthy donors”), operator training, validation of standard operating procedures, and equipment calibration the potential impact on inter-assay variability inherent to longitudinal studies using whole blood samples can be reduced. In addition, additional cell surface markers may be required to fully explore the impact of the inherent biological variation in the relative frequency of immune cell populations in functional assays using whole blood samples. For example, granulocyte populations present in relatively high frequencies in whole blood are absent in isolated PBMCs. Although we did not observe a correlation between granulocyte frequency and NK cell function (IFNγ) in our patient samples, the activation of the large and variable number of granulocytes present in whole blood samples may impact results depending on the immunological phenotypes and target cell populations of interest.

## Troubleshooting

Antibodies need to be titrated using whole blood protocols to ensure appropriate staining. Stimulation incubation times may have to be adjusted for different signaling proteins depending on the target (15–30 min is a good range to test). Mix FACS lyse/fix buffer and blood vigorously to ensure lysis of RBCs; if RBCs are left in the pellet the lyse/fix step can be repeated a second time. Golgiplug (Brefaldin A) may be added with 4 vs. 2 h of incubation time remaining to quantify intracellular cytokine production. Intracellular staining can be done in falcon tubes instead of a 96-well v-bottom plate, but the pellet is easier to visualize in a plate. Samples can be resuspended in flow buffer for up to 24 h or 1% PFA for longer storage at 4°C. CD45 and CD14 staining are not necessary but make gating on the CD56^+^ CD3^−^ population cleaner.

## Discussion

The workflow of some immune cell studies may be more compatible with protocols utilizing cryopreserved samples, for example multi-institute studies, however, due to the advantages reported here we suggest that some assays may be significantly improved through the implementation of whole blood protocols. These assays circumvent the limitations associated with the use of cryopreserved PBMCs, namely manipulation of cells and the thawing process which may alter cell phenotype and function. Consistent with previously reported differences between cryopreserved and whole blood PBMC assays, we show that cryopreservation results in an aberrant NK cell phenotype (24, 33, 34). We suggest that this discrepancy may lead to misinterpreted conclusions about altered immune cell phenotype and function. As a possible solution, we present methodologies for parallel assessment of immune cell receptor expression, signaling protein activity, and cytokine production in whole blood-derived NK cells. We have demonstrated the feasibility of these assays through the detection of target protein expression in both healthy and disease states (namely solid malignancies), the reproducibility of these assays in patient cohorts despite inherent inter-patient heterogeneity, and the validity of these assays in that our results are comparable to those previously described in the literature (2, 12, 32, 34, 39–44). They are simple, time-efficient, and allow for the assessment of any peripheral immune cell population using a minimal volume of whole blood. Finally, we suggest that they could be used to assess immune cell phenotype and function in any pathological condition, provided sufficient blood volumes.

## Data Availability Statement

The datasets for this study can be made available upon reasonable request.

## Ethics Statement

The studies involving human participants were reviewed and approved by Ottawa Health Science Research Ethics Board. The patients/participants provided their written informed consent to participate in this study.

## Author Contributions

MM and GT performed the experiments and data analysis and were responsible for manuscript preparation. JN and MS screened and consented all patients and performed blood processing on cryopreserved samples. MM, GT, CS, MK, and RA oversaw experimental design and data interpretation.

## Conflict of Interest

The authors declare that the research was conducted in the absence of any commercial or financial relationships that could be construed as a potential conflict of interest.
