# Supplementary material for: A Method of Assessment of Human Natural Killer Cell Phenotype and Function in Whole Blood
Source: Front Immunol. 2020 May 20;11:963. doi: 10.3389/fimmu.2020.00963 (PMC7251181; doi:10.3389/fimmu.2020.00963)
Supplement: Supplementary file 1 [file Data_Sheet_1.docx]

A Method of Assessment of Human Natural Killer Cell Phenotype and Function in Whole Blood

**Table 1.  Percentage and Median Fluorescence Intensity of NK Cell Surface Receptors.**

|  | **% positive CD56^Dim^ CD3- cells (median, IQR)** | | **MFI in CD56^Dim^ CD3-**  **(median, IQR)** | | |  |
| --- | --- | --- | --- | --- | --- | --- |
| **Target** | **Healthy** | **Cancer** | **Healthy** | **Cancer** | |  |
| **NKG2D** | 90.8 (87.1-92.9) | 88.1 (83.5-91.4) | 306.5 (260.8-388.5) | | 275.5 (225.3-296.0) | |
| **DNAM-1** | 76.2 (65.3-89.3) | 84.4 (75.9-87.9) | 179.5 (156.0-212.8) | | 193.0 (155.5-216.0) | |
| **NKG2A** | 34.6 (26.7-41.4) | 28.0 (21.8-40.2) | 21.5 (17.2-31.9) | | 20.8 (15.5-38.2) | |
| **PD-1** | 1.1 (0.6-1.9) | 1.0 (0.3-1.5) | 45.2 (38.8-52.38) | | 42.8 (38.9-50.6) | |
| **TIGIT** | 31.3 (28.9-43.0) | 35.0 (25.1-42.8) | 279.5 (240.5-294.5) | | 273.0 (212.5-302.3) | |
| **TIM-3** | 40.7 (22.4-46.5) | 38.4 (32.7-49.0) | 334.5 (286.5-394.8) | | 306.5 (244.8-361.5) | |
| **CD25** | 4.7 (4.0-7.5) | 7.0 (6.1-7.5) | 83.5 (63.4-171.5) | | 65.3 (50.50-176.0) | |
| **CD122** | 95.7 (93.7-97.2) | 94.3 (92.0-96.0) | 501.0 (380.5-654.5) | | 466.0 (307.0-666.5) | |
| **CD132** | 61.7 (42.3-71.3) | 39.1 (38.1-60.9) | 179.0 (132.0-207.0) | | 190.0 (180.0-192.0) | |
| **CD212** | 44.8 (32.6-55.6) | 52.6 (41.4-58.0) | 191.0 (180.5-201.0) | | 186.0 (157.0-199.0) | |

|  | **% positive CD56^Bright^ CD3- cells (median, IQR)** | | **MFI in CD56^Bright^ CD3-**  **(median, IQR)** | | |  |
| --- | --- | --- | --- | --- | --- | --- |
| **Target** | **Healthy** | **Cancer** | **Healthy** | **Cancer** | |  |
| **NKG2D** | 91.4 (87.1-93.5) | 84.4 (75.9-87.9) | 300.5 (255.8-341.0) | | 278.5 (207.3-353.3) | |
| **DNAM-1** | 73.7 (61.4-89.3) | 88.1 (83.5-91.4) | 275.0 (242.8-321.5) | | 252.5 (219.8-311.0) | |
| **NKG2A** | 38.0 (32.2-52.3) | 28.0 (21.8-40.2) | 150.0 (106.8-180.8) | | 88.2 (49.3-162.0) | |
| **PD-1** | 1.2 (0.6-2.2) | 1.0 (0.3-1.5) | 40.2 (33.1-46.8) | | 38.9 (31.6-42.8) | |
| **TIGIT** | 31.3 (26.8-43.0) | 35.0 (25.1-42.8) | 165.5 (123.8-188.8) | | 144.0 (113.0-172.3) | |
| **TIM-3** | 35.6 (22.0-41.6) | 38.4 (32.7-49.0) | 770.0 (614.5-948.8) | | 676.0 (503.5-783.8) | |
| **CD25** | 18.5 (15.3-27.0) | 23.9 (18.9-32.0) | 152.0 (104.2-224.5) | | 111.0 (74.4-224.0) | |
| **CD122** | 97.5 (96.0-98.5) | 96.6 (92.1-98.0) | 694.0 (542.0-860.0) | | 625.0 (377.8-1275.3) | |
| **CD132** | 80.4 (59.4-95.6) | 76.3 (72.1-86.0) | 223.0 (179.0-262.0) | | 229.0 (213.0-232.0) | |
| **CD212** | 18.6 (13.0-28.8) | 39.3 (11.5-43.0) | 345.0 (322.5-375.5) | | 321.0 (257.0-347.0) | |

**Table 2.  Percentage and Median Fluorescence Intensity of NK Cell Activating/ Inhibitory/ Cytokine Receptors Measured in Cryopreserved PBMCs.**

|  | **% positive CD56^Dim^ CD3- cells (median, IQR)** | | **MFI in CD56^Dim^ CD3-**  **(median, IQR)** | | |  |
| --- | --- | --- | --- | --- | --- | --- |
| **Target** | **Cryopreserved** | **Whole Blood** | **Cryopreserved** | **Whole Blood** | |  |
| **NKG2D** | 69 (57.1-82.5) | 84.4 (75.9-87.9) | 69 (57.1-82.5) | | 84.4 (75.9-87.9) | |
| **DNAM-1** | 13.6 (8.8-17) | 88.1 (83.5-91.4) | 13.6 (8.8-17) | | 88.1 (83.5-91.4) | |
| **NKG2A** | 78.6 (56.8-83.2) | 28.0 (21.8-40.2) | 78.6 (56.8-83.2) | | 28.0 (21.8-40.2) | |
| **PD-1** | 25.6 (13.5-39.2) | 1.0 (0.3-1.5) | 25.6 (13.5-39.2) | | 1.0 (0.3-1.5) | |
| **TIGIT** | 90.8 (71.2-96.5) | 35.0 (25.1-42.8) | 90.8 (71.2-96.5) | | 35.0 (25.1-42.8) | |
| **TIM-3** | 41.5 (38.1-55.1) | 38.4 (32.7-49.0) | 41.5 (38.1-55.1) | | 38.4 (32.7-49.0) | |
| **CD25** | 10.3 (6.6-16.7) | 23.9 (18.9-32.0) | 10.3 (6.6-16.7) | | 23.9 (18.9-32.0) | |
| **CD212** | 38.4 (32.3-44.1) | 39.3 (11.5-43.0) | 38.4 (32.3-44.1) | | 39.3 (11.5-43.0) | |

|  | **% positive CD56^Bright^ CD3- cells (median, IQR)** | | **MFI in CD56^Bright^ CD3-**  **(median, IQR)** | | |  |
| --- | --- | --- | --- | --- | --- | --- |
| **Target** | **Cryopreserved** | **Whole Blood** | **Cryopreserved** | **Whole Blood** | |  |
| **NKG2D** | 82.9 (75.4-87) | 84.4 (75.9-87.9) | 82.9 (75.4-87) | | 84.4 (75.9-87.9) | |
| **DNAM-1** | 23.9 (15.7-36.6) | 88.1 (83.5-91.4) | 23.9 (15.7-36.6) | | 88.1 (83.5-91.4) | |
| **NKG2A** | 84.9 (77.7-88.5) | 28.0 (21.8-40.2) | 84.9 (77.7-88.5) | | 28.0 (21.8-40.2) | |
| **PD-1** | 27 (15.4-39.2) | 1.0 (0.3-1.5) | 27 (15.4-39.2) | | 1.0 (0.3-1.5) | |
| **TIGIT** | 80.1 (55.4-95.7) | 35.0 (25.1-42.8) | 80.1 (55.4-95.7) | | 35.0 (25.1-42.8) | |
| **TIM-3** | 48.3 (38.9-66.0) | 38.4 (32.7-49.0) | 48.3 (38.9-66.0) | | 38.4 (32.7-49.0) | |
| **CD25** | 8.2 (3.2-13.0) | 23.9 (18.9-32.0) | 8.2 (3.2-13.0) | | 23.9 (18.9-32.0) | |
| **CD212** | 22.1 (8.8-32.7) | 39.3 (11.5-43.0) | 22.1 (8.8-32.7) | | 39.3 (11.5-43.0) | |

**Table 3. Median Fluorescence Intensity of Intracellular Signaling Molecules.**

|  | **MFI in CD56^Bright^ CD3- (median, IQR)** | |
| --- | --- | --- |
| **Target** | **Healthy** | **Cancer** |
| **STAT5** | 841.0 (683.0-880.0) | 800.0 (755.0-883.0) |
| **STAT4** | 767.0 (534.0-1021.5) | 595.0 (477.0-691.0) |
| **p38 MAPK** | 799.0 (671.5-996.0) | 972.0 (829.0-1051.0) |
| **S6** | 691.0 (615.0-883.0) | 912.5 (828.0-973.8) |

|  | **MFI in CD56^Dim^ CD3- (median, IQR)** | |
| --- | --- | --- |
| **Target** | **Healthy** | **Healthy** |
| **STAT5** | 642.0 (573.0-728.0) | 642.0 (573.0-728.0) |
| **STAT4** | 487.0 (340.5-667.5) | 487.0 (340.5-667.5) |
| **p38 MAPK** | 728.0 (622.5-918.5) | 728.0 (622.5-918.5) |
| **S6** | 578.0 (489.0-625.0) | 578.0 (489.0-625.0) |

**Table 4. Intracellular and Extracellular Measurements of IFNγ.**

|  | **% positive CD56^Bright^ CD3- cells (median, IQR)** | |
| --- | --- | --- |
| **Stimulation** | **Healthy** | **Cancer** |
| **PMA-ionomycin** | 11.2 (8.1-14.1) | 11.8 (2.8-16.4) |
| **IL-2/12** | 18.1 (13.9-31.9) | 16.8 (11.0-33.1) |
|  | **IFNγ (pg/mL) (median, IQR)** | |
| **Stimulation** | **Healthy** | **Cancer** |
| **PMA-ionomycin** | 737.3 (268.2-5000.0) | 516.2 (284.3-1069.7) |
| **IL-2/12** | 1596.1 (185.9-2234.3) | 1427.8 (570.9-2281.8) |

|  | **% positive CD56^Dim^ CD3- cells (median, IQR)** | |
| --- | --- | --- |
| **Stimulation** | **Healthy** | **Cancer** |
| **PMA-ionomycin** | 6.3 (3.9-8.0) | 6.0 (4.7-13.5) |
| **IL-2/12** | 8.4 (3.9-17.1) | 4.2 (3.9-23.6) |
|  | **IFNγ (pg/mL) (median, IQR)** | |
| **Stimulation** | **Healthy** | **Cancer** |
| **PMA-ionomycin** | 737.3 (268.2-5000.0) | 516.2 (284.3-1069.7) |
| **IL-2/12** | 1596.1 (185.9-2234.3) | 1427.8 (570.9-2281.8) |

**Supplemental Figure 1. Gating strategies for target receptors and IFNγ gating.**

Matched antibody isotype controls were used to set the gates for quantification of NK cell receptors NKG2D, PD-1, DNAM-1, NKG2A, TIGIT, TIM-3, CD25, CD212, CD122, and CD132. Gating to assess intracellular IFNγ was set based on matched unstimulated control samples.

**Supplemental Figure 2. Cryopreserved PBMC gating strategy.**

After Ficoll density centrifugation, PBMCs were isolated, washed, and stored in liquid nitrogen in 90% FBS 10% DMSO. PBMCs were thawed, rested overnight, and stained using a standard protocol. Debris, doublets, and dead cells were excluded before gating on CD14^-^ CD56^Bright/Dim^ CD3^-^ cells to assess activating/ inhibitory receptor and cytokine receptor expression based on isotype staining.

**Supplemental Figure 3. STAT4 phosphorylation corresponds to IFNγ expression.**

The IFNγ MFI of CD56^Bright/Dim^ CD3^-^ cells was plotted against intracellular pSTAT4 MFI from the same patient samples. A correlation was found between signalling protein expression and NK cell function (IFNγ expression intensity as proxy) upon performing a linear regression in both of the NK cell populations (p<0.05).

**Supplemental Figure 4. Proportion of granulocytes is not related to NK cell IFNγ production.**

Cells were gated on to exclude debris, doublets, dead cells, and CD14^+^ cells prior to gating on SSC-A^hi^ FSC-A^hi^ cells. (A) The percentage of SSC-A^hi^ FSC-A^hi^ cells was plotted against intracellular IFNγ produced by the same patient samples. No correlation was found upon performing a linear regression.
