# Supplementary material for: A Method of Assessment of Human Natural Killer Cell Phenotype and Function in Whole Blood
Source: Front Immunol. 2020 May 20;11:963. doi: 10.3389/fimmu.2020.00963 (PMC7251181; doi:10.3389/fimmu.2020.00963)
Supplement: Supplementary file 2 [file Data_Sheet_2.docx]

## Appendix

## Protocol 1 – Extracellular Receptor Staining (Figure 1A)

Objective: Assess cell surface receptor expression in Natural Killer cells from whole blood.

1. Collect whole blood in sodium-heparin tubes (blood drawn by trained nurse/ phlebotomist)
2. Prepare FACS lyse/ fix buffer in advance (1:5 dilution with diH_2_O; 20X blood volume per sample)
3. Transfer 200 μL of whole blood to 15 mL falcon tube
4. Add extracellular staining (ECS) mix (40 μL); Mix by pipetting up and down
5. Incubate for 15 mins at room temperature
6. Add FACS lyse/ fix buffer
7. Incubate for 10 minutes in 37°C water bath
8. Spin at 500 g for 8 minutes
9. Aspirate supernatant and resuspend in 1 mL flow buffer
10. Spin at 500 g for 5 mins
11. Resuspend in 200 μL flow buffer or 1% PFA
12. Store at 4°C for up to 24 hours (FB) or 72 hours (PFA)

## Protocol 2 – Intracellular Signaling Protein Phosphorylation Staining (Figure 4A)

Objective: Assess signaling protein/ transcription factor phosphorylation in response to stimuli in Natural Killer cells from whole blood.

1. Collect whole blood in sodium-heparin tubes (blood drawn by trained nurse/ phlebotomist)
2. Prepare FACS lyse/ fix buffer in advance (1:5 dilution with diH_2_O; 20X blood volume per sample)
3. Aliquot 500 μL blood into new sodium-heparin tubes
4. Add stimulation and ECS mix and incubate for 20 minutes in 37°C water bath
5. Transfer whole blood to 15 mL falcon tubes
6. Add FACS lyse/fix buffer
7. Incubate for 10 minutes in 37°C water bath
8. Spin at 500 g for 8 minutes
9. Aspirate supernatant and wash with 1 mL flow buffer
10. Spin at 500 g for 5 minutes
11. Aspirate supernatant and resuspend in 500 μL chilled BD Perm III buffer
12. Incubate on ice in the dark for 30 minutes
13. Spin at 300 g for 10 minutes
14. Aspirate supernatant and resuspend in 400 μL flow buffer
15. Transfer 200 μL/well into 96 well v-bottom plate
16. Spin at 500 g for 5 minutes
17. Empty plate and resuspend in appropriate intracellular staining (ICS) mix (200 μL)
18. Incubate at room temperature in the dark for 1 hour
19. Spin at 500 g for 5 mins
20. Empty plate and resuspend in 200 μL flow buffer 1% PFA
21. Store at 4°C for up to 24 hours (FB) or 72 hours (PFA)

## Protocol 3 – Intracellular IFNγ Staining (Figure 5A)

Objective: Quantify intracellular IFNγ production as a measure of activity in Natural Killer cells from whole blood.

1. Collect whole blood in sodium-heparin tubes (blood drawn by trained nurse/ phlebotomist)
2. Prepare FACS lyse/ fix buffer in advance (1:5 dilution with diH_2_O; 20X blood volume per sample)
3. Aliquot 1 mL of whole blood into new sodium-heparin tubes
4. Incubate whole blood with PMA-ionomycin for 5 hours at 37°C and IL-2/IL-12 for 24 hours at 37°C
5. Add 10 ug/mL Golgi plug (Brefaldin A) per tube for the last 2 hours of each incubation
6. Invert tubes 10 times and incubate at 37°C for remaining 2 hours
7. Collect 600 μL whole blood in Eppendorf tube, spin at 13000 rpm for 1 minute, and store at -80°C for IFNγ ELISA
8. Transfer remaining 400 μL to 15mL falcon tubes
9. Incubate with Fc block (50 μL) for 5 mins at room temperature
10. Add ECS mix (40 μL); Mix by pipetting up and down
11. Incubate for 15 mins at room temperature
12. Add FACS lyse/ fix buffer
13. Incubate for 10 minutes in 37°C water bath
14. Spin at 500 g for 8 minutes
15. Aspirate supernatant and resuspend in 1 mL flow buffer
16. Spin at 500 g for 5 mins
17. Aspirate supernatant and resuspend in 500 μL chilled BD Perm III buffer
18. Incubate on ice in the dark for 30 minutes
19. Spin at 300 g for 10 minutes
20. Aspirate supernatant and resuspend in 400 μL flow buffer
21. Transfer 200 μL/ well into 96 well v-bottom plate
22. Spin at 500 g for 5 minutes
23. Empty plate and resuspend in appropriate ICS mix (200 μL)
24. Incubate at 4°C for 30 minutes
25. Empty plate and resuspend in 200 μL flow buffer or 1% PFA
26. Store at 4°C for up to 24 hours (FB) or 72 hours (PFA)
